# β-Hydroxybutyric Inhibits Vascular Calcification via Autophagy Enhancement in Models Induced by High Phosphate

**DOI:** 10.3389/fcvm.2021.685748

**Published:** 2021-08-20

**Authors:** Jianwen Liang, Jieping Huang, Wanbing He, Guangzi Shi, Jie Chen, Hui Huang

**Affiliations:** ^1^Department of Cardiology, Sun Yat-sen Memorial Hospital, Sun Yat-sen University, Guangdong, China; ^2^Department of Cardiology, the Eighth Affiliated Hospital, Sun Yat-sen University, Guangdong, China; ^3^Department of Radiology, Sun Yat-Sen Memorial Hospital, Sun Yat-Sen University, Guangdong, China; ^4^Department of Radiation Oncology, Sun Yat-sen Memorial Hospital, Sun Yat-sen University, Guangdong, China

**Keywords:** vascular calcification, β-hydroxybutyric acid, metabolic abnormalities, osteogenic phenotypic differentiation, vascular smooth muscle cells

## Abstract

**Background:** Vascular calcification (VC) is a landmark of aging, while β-hydroxybutyric acid (BHB) induced by calorie restriction has been identified as a promising factor to extend the lifespan. However, the effect of BHB on VC and the potential mechanism remain unknown.

**Methods:** A total of 160 subjects with or without metabolic abnormalities (MAs) were assigned to four groups according to different calcification severities. The association between BHB, MAs, and VC was investigated *via* mediation analysis. Then, with high phosphate-induced calcification models, the effect of BHB on arterial ring calcification and osteogenic phenotypic differentiation of vascular smooth muscle cells (VSMCs) was investigated. Hereafter the expressions of autophagy biomarkers, autophagy flux, and effects of autophagy inhibitors on VC were detected.

**Results:** Severe VC was observed in the elderly, accompanied with a higher proportion of hypertension, chronic kidney disease, and lower estimated glomerular filtration rate. The serum BHB level was an independent influencing factor of VC severities. With mediation analysis, BHB was determined as a significant mediator in the effects of MAs on VC, and the indirect effect of BHB accounted for 23% of the total effect. Furthermore, BHB directly inhibited arterial ring calcification and osteogenic phenotypic differentiation in VSMCs, accompanied with autophagy enhancement in VSMCs. In accordance, the inhibition of autophagy counteracted the protective effect of BHB on VC.

**Conclusion:** The present study demonstrated that BHB mediated the effects of MAs on VC; then, it further elucidated that BHB could inhibit arterial and VSMC calcification *via* autophagy enhancement.

## Introduction

Vascular calcification (VC), characterized by the accumulation of calcium phosphate crystals and the phenotypical transition of vascular smooth muscle cells (VSMCs) inside the vessels, is related to a variety of diseases, such as diabetes, chronic kidney disease (CKD), atherosclerosis, and other cardiovascular diseases (1–3). Although the incidence of VC remains high in clinics, there are still no approved or substantially effective therapies for the treatment of VC. Therefore, exploring the novel therapy for VC has been an important tough problem waiting to be solved.

It has become increasingly clear that metabolic syndrome (METs) is a major risk factor of VC (4, 5). Emerging evidence has demonstrated that calorie restriction (CR), a nutritional intervention of reduced energy intake but with adequate nutrition, effectively regulates the metabolic balance, ameliorates metabolic abnormalities (MAs), and reduces VC (6–9). Meanwhile, β-hydroxybutyric acid (BHB), which could be generated by CR, has been proven to promote autophagy and prevent VSMC senescence (10). However, whether BHB can prevent arterial calcification in populations with MAs or VC and the potential mechanism remain unclear.

Among the numerous etiologies, autophagy, an important biological process that contributes to cellular homeostasis, has been recognized as an important influencing factor of survival and function in VSMCs in the process of VC (11, 12). Autophagy exists in three separate forms: microautophagy, chaperone-mediated autophagy, and macroautophagy (13). The term autophagy usually refers to macroautophagy, which is the most prevalent and the only form of autophagy mentioned here. Alterations in autophagy have been documented in VSMCs in response to various stimuli, resulting in the modulation of VSMC functions, including VC (14, 15). On the contrary, the activation of autophagy stimulates VSMC survival, maintains normal vascular cell function, and protects against VC (16, 17). Notably, the accumulated evidence has confirmed that BHB plays a broad role in regulating longevity and affects the development of aging (18). Hence, it may discreetly propose that BHB intervention may protect VC and the underlying mechanism is related to autophagy.

Based on the uncertainties mentioned above, we hypothesized that insufficient autophagy is associated with VC, and autophagy enhancement induced by BHB maintains the VSMC function and protects against VC. To address these assumptions, the role of BHB in the correlation of MAs and VC was investigated, and then the effect of BHB administration on high phosphate-induced VC models and the potential mechanism related to autophagy were further explored. This study may provide herein novel insights into the mechanism of autophagy in VC and shed novel light on the promising therapeutic target of the early prevention of VC.

## Methods

### Subjects

This study was designed to investigate the relations between BHB and calcification under different metabolic status. The study recruited 160 patients from the Affiliated Hospitals of Sun Yat-sen University from January 2015 to July 2020. The participants, ages between 42 and 91, all underwent chest computerized tomographic (CT) scan and serum β-hydroxybutyric acid measurement and had no missing data on any components of the METs. Patients with severe diseases that might affect nutrient absorption and energy metabolism were excluded, including inflammatory bowel disease, cachexia, cirrhotic hepatic disease, malignant tumor, *etc*. Demographic data and medical history were acquired using standard questionnaires. The trial was conducted according to the International Conference on Harmonization—Good Clinical Practice guidelines and the Declaration of Helsinki. All patients were enrolled after informed consents were obtained.

### Computerized Tomographic Analysis

All the patients underwent a non-enhanced chest CT scan to evaluate thoracic aorta calcification (TAC). As described by Agatston et al. (19), the calcium score of each lesion was calculated by multiplying the area of the calcified speck by the density factor derived from the maximal Hounsfield units within this area (20). The grouping criteria of TAC severity are as follows: TAC score = 0: no TAC; TAC score = 1–100: mild TAC; TAC score = 101–500: moderate TAC; and TAC score >500: severe TAC.

The chest CT scans were performed using the same equipment (SOMATOM Sensation 64 CT). The details of the scanning acquisition were described previously (21). Two experienced investigators, blinded to all clinical information, randomly analyzed all the CT images.

### Definition of the Metabolic Abnormalities

The definition of METs was diverse from each other, and developing a standard one has been challenging. One of the best accepted definitions was provided by the US National Heart, Lung, and Blood Institute and the American Heart Association Consensus Statement (22). In this study, we referred to the METs definition and determined MAs with one and more of the following parameters: large waist circumference (women >80 cm and men >90 cm, according to the standard of abdominal obesity in the Chinese population), elevated triglycerides [≥1.7 mmol/L (150 mg/dl)], low high-density lipoprotein cholesterol (HDL-C) [men <1.03 mmol/L (40 mg/dl) and women <1.29 mmol/L (50 mg/dl)], impaired fasting glucose [≥6.1 mmol/L (110 mg/dl) or on antidiabetic medication), and elevated blood pressure (≥130/85 mmHg or self-reported use of medications for hypertension).

### Collection of Laboratory Parameters

Blood samples were collected from the patients in the morning after more than 10 h of fasting. The following parameters were tested or determined in the Institutional Central Laboratory of the Affiliated Hospital of Sun Yat-sen University: fasting glucose, total cholesterol, triglyceride (TG), high-density lipoprotein cholesterol (HDL-C), low-density lipoprotein cholesterol (LDLC), serum calcium (Ca), serum phosphate (Pi), and estimated glomerular filtration rate (eGFR). All the biochemical parameters were analyzed by using a standardized and certified TBA-120 autoanalyzer (Toshiba Medical Systems, Japan).

### Establishment and Examination of Calcification Models in Arterial Rings and VSMCs

The arterial rings and VSMCs were isolated or extracted as previously described (23). Calcification was induced by adding inorganic phosphate (Na_2_HPO_4_, Sigma, USA) to normal medium at a concentration of 2.6 mM (calcification medium). To evaluate the impact of BHB on calcification, BHB (Sigma, USA) was administrated into a calcification medium at a concentration of 4.0 mM (BHB intervention medium) (24). The arterial rings were cultured for 14 days, while VSMCs were cultured for 7 days, with the medium for both refreshed every 2 to 3 days (23).

To evaluate whether autophagy inhibition could reverse the protective effects of BHB on VC, the autophagosome inhibitor 3-methyladenine (Selleck, USA), at a concentration of 20 μM, and the autophagic flux inhibitor chloroquine (Selleck, USA), at a concentration of 10 μM, were administrated to the calcification models with BHB intervention (25).

### Von Kossa Staining and Alizarin Red S Staining

Von Kossa staining was used to detect the calcified depositions of arterial rings. The staining experiments were performed as previously described (25). The calcium deposits displayed black or dark brown colors under the microscope.

For Alizarin Red S staining, VSMCs in six-well plates were washed three times with phosphate-buffered saline (PBS) after the indicated treatments and then fixed with 4% paraformaldehyde for 30 min. After that, the fixed VSMCs were washed and then stained with 2% Alizarin Red S solution (Servicebio, China) for 10 min. The stained calcium deposits were observed and photographed under a microscope. The area positive for Alizarin Red S staining displayed a red color.

### Calcium Assay

VSMCs were washed three times with PBS and then decalcified with 0.6 M hydrochloric acid for 12 h at 4°C. The supernatant was collected, of which the calcium concentration was determined as previously described (25).

### Quantitative Real-Time Polymerase Chain Reaction

Total RNA was extracted, reverse-transcribed, and amplified to detect some specific mRNA transcription as previously described (26). The primers were designed on Primerbank and verified with primer-BLAST on PubMed. The PCR primers were as follows:

GAPDH, forward 5′-AGGTCGGTGTGAACGGATTTG-3′ and reverse 5′-GGGGTCGTTGATGGCAACA-3′; Runx2, forward 5′-GACTGTGGTTACCGTCATGGC-3′ and reverse 5′-ACTTGGTTTTTCATAACAGCGGA-3′; BMP-2, forward 5′-GGGACCCGCTGTCTTCTAGT-3′ and reverse 5′-TCAACTCAAATTCGCTGAGGAC-3′; SM22a, forward 5′-CCAACAAGGGTCCATCCTACG-3′ and reverse 5′-ATCTGGGCGGCCTACATCA-3′; α-SMA, forward 5′-CCCAGACATCAGGGAGTAATGG-3′ and reverse 5′-TCTATCGGATACTTCAGCGTCA-3′.

The qPCR experiments were independently repeated three times, and the quantification was performed using the ΔΔCt method. The results were normalized to GAPDH transcription.

### Western Blot Analysis

VSMCs were washed three times with PBS and collected after treatment with RIPA lysis buffer as described previously (26). The proteins were boiled, and the same amount of proteins was subjected to SDS-PAGE and transferred onto polyvinylidene difluoride membranes (Merck-Millipore, the USA). The membranes were incubated successively with 5% bovine serum albumin for 1 h and then stained with primary antibodies for 12 h at 4°C, including anti-smoothelin (1:1,000 dilution, Abcam, USA), anti-Runx2 (1:500 dilution, CST, USA), anti-BMP2 (1:1,000 dilution, Novus, USA), anti-GAPDH (1:1,000 dilution, CST, USA), and anti-SM22α (1:1,000 dilution, Abcam, USA). The membranes were then incubated with secondary goat anti-rabbit IgG antibody (1:5,000 dilution, Santa Cruz, USA) for 1 h. Finally, the reaction was visualized by a chemiluminescence image system (Proteinsimple, USA), and the density of the bands was semi-quantified *via* the image software Image J (National Institutes of Health, *V* 1.53, USA).

### Autophagy Detection Using mRFP-GFP-LC3 Adenoviral Vector

mRFP-GFP-LC3 double-labeled adenovirus was purchased from HanBio Technology Co., Ltd., and adenoviral infection was performed according to the instructions of the manufacturer (25). Isolated mouse VSMCs were plated in six orifice plates, and the cells were transduced with adenovirus after reaching 50% confluence by Dulbecco's modified Eagle's medium supplemented with 5% fetal bovine serum (FBS) for 24 h at 37°C. After infection, the VSMCs were then grown in a medium with 2.5% FBS for 24 h before changing to a complete culture medium administrated with 2.6 mM Pi and 4.0 mM BHB. Autophagy was observed through a fluorescence microscope (Nikon Eclipse Tis). Autophagic flux was determined by evaluating the number of GFP and RFP puncta (puncta/cell was counted).

### Statistical Analysis

Data of the continuous variables are presented as mean and standard deviation (SD), skewed data as median (25th and 75th percentiles), and categorical variables as absolute numbers and percentages. Comparisons among groups were attained by one-way ANOVA (for distributed variables) or chi-square test (for categorical variables). For skewed data, the cases were ranked as new variables, and then an analysis of variance was performed.

Regression analysis was performed to examine whether BHB concentration was an independent influencing variable for TAC severities. A univariate linear regression model (model 1) was initially analyzed. Then, model 1 was adjusted with significant and potential confounding variables, which formed multivariate linear regression models (models 2 and 3), to further investigate the association between BHB and TAC severities. Specifically, model 2 was adjusted with age, sex, BMI, waist circumference, hypertension (HTN), and type 2 diabetes mellitus (T2DM), while model 3 was adjusted with age, sex, BMI, waist circumference, HTN, T2DM, TG, HDL, eGFR, Ca, and Pi.

Mediation analysis was conducted on SPSS 22.0 with model 4 in PROCESS plugin, which was introduced by (27). Before the analysis, we hypothesized that MAs (the independent variable) influenced TAC severities (dependent variable) directly as well as indirectly *via* BHB (mediator variable). Mediation analysis was used to validate the hypothesis, which was composed of three steps. Firstly, the direct effect of MAs on TAC severities (*c*′) was evaluated. Secondly, the effect of MAs on serum BHB level (*a*) was analyzed. Thirdly, the indirect effect of MAs on TAC severities *via* serum BHB level (*a* ^*^ *b*) was evaluated, while *b* referred to the effect of BHB on TAC severities controlling for MAs. The sum of the direct effect and the indirect effect was considered as the total effect of MAs on TAC severities (*c*), meaning that *c* = *c*′ + *a* ^*^ *b*. In this research, age was included as a control variable. The effects were significant if their 95% confidence interval (CI) did not cross zero (28). BHB would be determined as a mediating factor if the results showed that both the total effect and the indirect effect were evident.

We used IBM SPSS Statistics 22.0 for all data analyses, and a two-tail *P* < 0.05 has been considered significant after Bonferroni correction.

## Results

### Baseline Characteristics

A total of 160 participants (69.4% are female; ages between 42 and 91) were finally enrolled within the specified time, and the baseline characteristics of the subjects by different degrees of TAC are described in Table 1. The severity of TAC increased with aging; in addition, a higher occurrence of hypertension and CKD and a lower eGFR were observed in the severe TAC group. Meanwhile, no significant differences were found in gender, glucolipid, and Ca/Pi metabolism between groups.

**Table 1:** Clinical characteristics of all participants according to different severities of TAC.

	**No TAC (*n* = 43)**	**Mild TAC (*n* = 35)**	**Moderate TAC (*n* = 32)**	**Severe TAC (*n* = 50)**
Age, year	52 (49, 60)	62 (56, 66)^***^	64 (57, 68)^***^	72 (62, 79)^***^^###^^&^
Male, *n* (%)	11 (25.6)	11 (31.4)	9 (28.1)	18 (36.0)
BMI, kg/m^2^	23.27 (20.31, 25.94)	21.40 (18.97, 23.90)	23.33 (21.69, 24.67)	23.03 (20.12, 25.31)
WC, cm	78 (73.50, 81.00)	77.00 (72.75, 83.25)	79.00 (73.00, 84.00)	77.00 (70.25, 84.00)
HTN, *n* (%)	8 (21.6)	10 (30.3)	10 (32.3)	30 (63.8)^***^^##^^&^
T2DM, *n* (%)	6 (15.4)	7 (20.6)	3 (10.0)	16 (32.0)
CKD, *n* (%)	19 (47.5)	23 (65.7)	24 (75.0)	45 (90.0)^***^^###^
FBG, mmol/L	5.08 ± 1.06	5.30 ± 1.30	5.45 ± 2.55	5.81 ± 2.14
TC, mmol/L	4.95 ± 1.23	4.92 ± 1.40	5.05 ± 1.37	5.01 ± 1.54
HDL-C, mmol/L	1.24 (1.03, 1.46)	1.26 (1.00, 1.46)	1.15 (1.01,1.39)	1.27 (1.08, 1.47)
LDL-C, mmol/L	2.84 (2.45, 3.31)	2.59 (2.18, 3.59)	3.16 (2.63, 3.85)	2.99 (2.12, 3.93)
TG, mmol/L	1.22 (0.91, 1.66)	1.27 (0.96, 1.76)	1.34 (0.92, 1.81)	1.38 (0.87, 1.32)
eGFR, ml/min/1.73 m^2^	90.00 (55.23, 100.20)	81.87 (73.42, 92.5)	75.64 (65.77, 89.80)	65.06 (44.56, 80.16)^***^^##^
Ca, mmol/L	2.27 (2.19, 2.35)	2.22 (2.13, 2.32)	2.28 (2.13, 2.35)	2.22 (2.13, 2.32)
Pi, mmol/L	1.15 (1.08, 1.28)	1.15 (1.02, 1.24)	1.17 (1.02, 1.24)	1.14 (1.04, 1.33)
TAC score	0	19.90 (5.6, 59.6)	255.10 (194.3, 353.1)	1,812.10 (800.1, 7164.4)

*Data are presented as mean ± SD or median (25th and 75th percentiles). HTN, T2DM, and CKD are presented as n (%). Compared with the normal group*:

*** *P < 0.001; compared with the mild TAC group*:

## *P < 0.01, and*

### *P < 0.001; compared with the moderate TAC group*:

& *P < 0.05*.

*TAC, thoracic aorta calcification; BMI, body mass index; WC, waist circumference; HTN, hypertension; T2DM, type 2 diabetes mellitus; CKD, chronic kidney disease; FBG, fast blood glucose; TC, total cholesterol; HDL-C, high density lipoprotein cholesterol; LDL-C, low density lipoprotein cholesterol; TG, triglycerides; eGFR, estimated glomerular filtration rate; Ca, calcium; Pi, phosphate*.

### BHB Was Related With MAs and VC and Partly Mediated the Effects of MAs on VC

In this study, the patients with MAs had higher TAC scores than those without MAs [205.3 (4.7, 1,031.5) vs. 26.1 (0.0, 310.3), *P* < 0.01]. Furthermore, we investigated the relationship between serum BHB levels and TAC severities with linear regression analysis (Table 2). The results showed that, in the univariate linear regression (model 1), BHB was a protective factor for TAC. After adjusting for some potential influencing factors and metabolic parameters, including age, sex, WC, HTN, T2DM, TG, HDL-C, *etc*., the results still showed that BHB was an independent protective factor for TAC (*P* < 0.05).

**Table 2:** Linear regression analyses between BHB and TAC severities.

**Model**	**β**	**95%CI**	***P*-value**	**Adjusted *R^**2**^***
Model 1	−0.260	(−0.491, −0.129)	0.001	0.062
Model 2	−0.256	(−0.562, −0.108)	0.004	0.326
Model 3	−0.234	(−0.545, −0.066)	0.013	0.307

*Model 1: unadjusted; model 2: adjusted with age, sex, BMI, WC, HTN, and T2DM; model 3: adjusted with age, sex, BMI, WC, HTN, T2DM, TG, HDL-C, eGFR, Ca, and Pi*.

*BHB, β-hydroxybutyric; BMI, body mass index; WC, waist circumference; HTN, hypertension; T2DM, type 2 diabetes mellitus; TG, triglycerides; HDL-C, high density lipoprotein cholesterol; eGFR, estimated glomerular filtration rate; Ca, calcium; Pi, phosphorus*.

**Figure 1:**
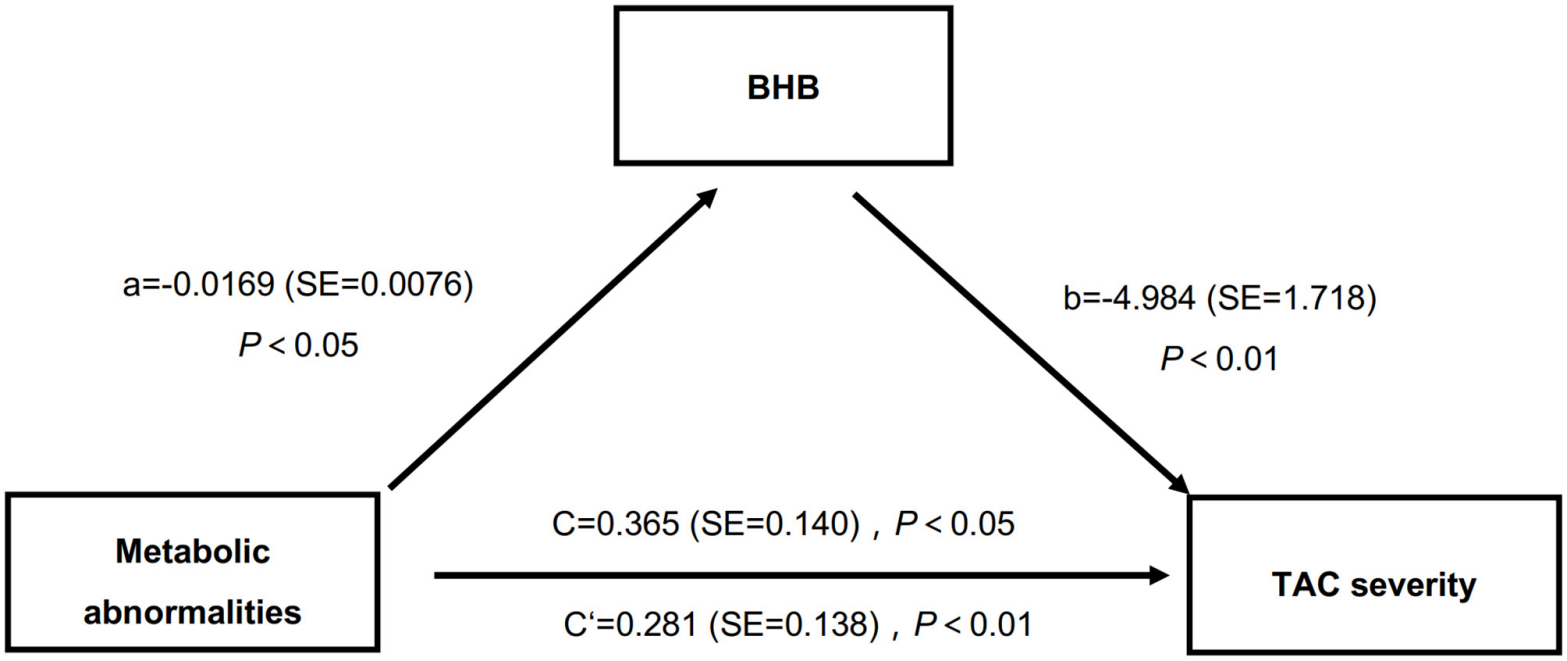
BHB partly mediated the relations between MAs and TAC severities. BHB, β-hydroxybutyric acid; MAs, metabolic abnormalities; TAC, thoracic aorta calcification.

It has been well-accepted that MAs contribute to the occurrence of VC, while BHB affected the metabolic status (4, 29). Here we performed a mediation analysis to further analyze whether BHB mediated the effects of MAs on TAC severities. The results showed that, in this study (Figure 1), the presence of MAs had a negative impact on serum BHB level (β = −0.0169, *P* < 0.05), and the BHB level also had a negative impact on TAC (β = −4.984, *P* < 0.01). Moreover, the presence of MAs had a significant positive effect on TAC severities (β = 0.365, *P* < 0.001), and after the mediator variable BHB was placed, the effect of MAs on TAC was still significant (β = 0.281, *P* < 0.05). In other words, MAs negatively affect BHB, while BHB partly mediated the effects of MAs on TAC. Specifically, BHB accounted for 23% of the association between MAs and TAC (direct effect: β = 0.281, 95%CI: 0.023–0.548; indirect effect: β = 0.084, 95%CI: 0.015–0.170).

### BHB Inhibited High Phosphate-Induced VC

**Figure 2:**
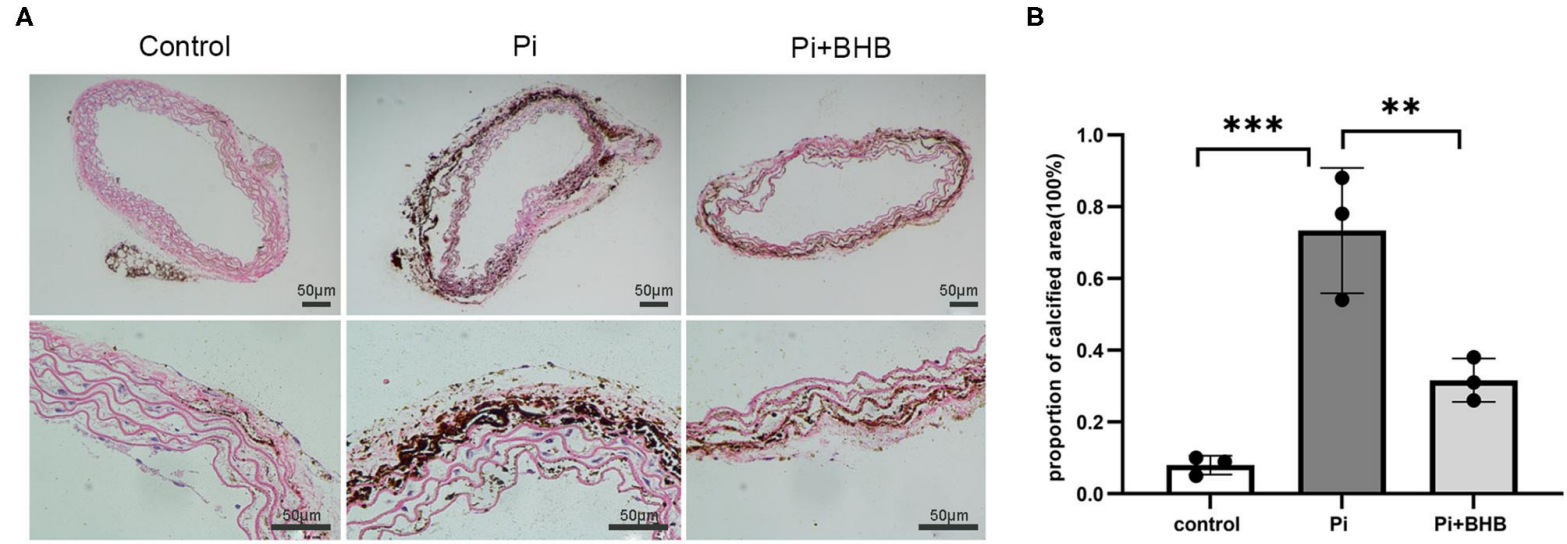
β-Hydroxybutyric acid ameliorated the Pi-induced arterial calcification. **(A)** Von Kossa staining of arterial rings. Pieces of mice aorta were cultured in calcification medium (Pi: 2.6 mM) for 14 days. Scale bar: 50 μm. **(B)** Proportion of calcium areas in the total cross-sectional aorta area. The analysis was performed by Image J. Pi, phosphate; **P < 0.01; ***P < 0.001.

**Figure 3:**
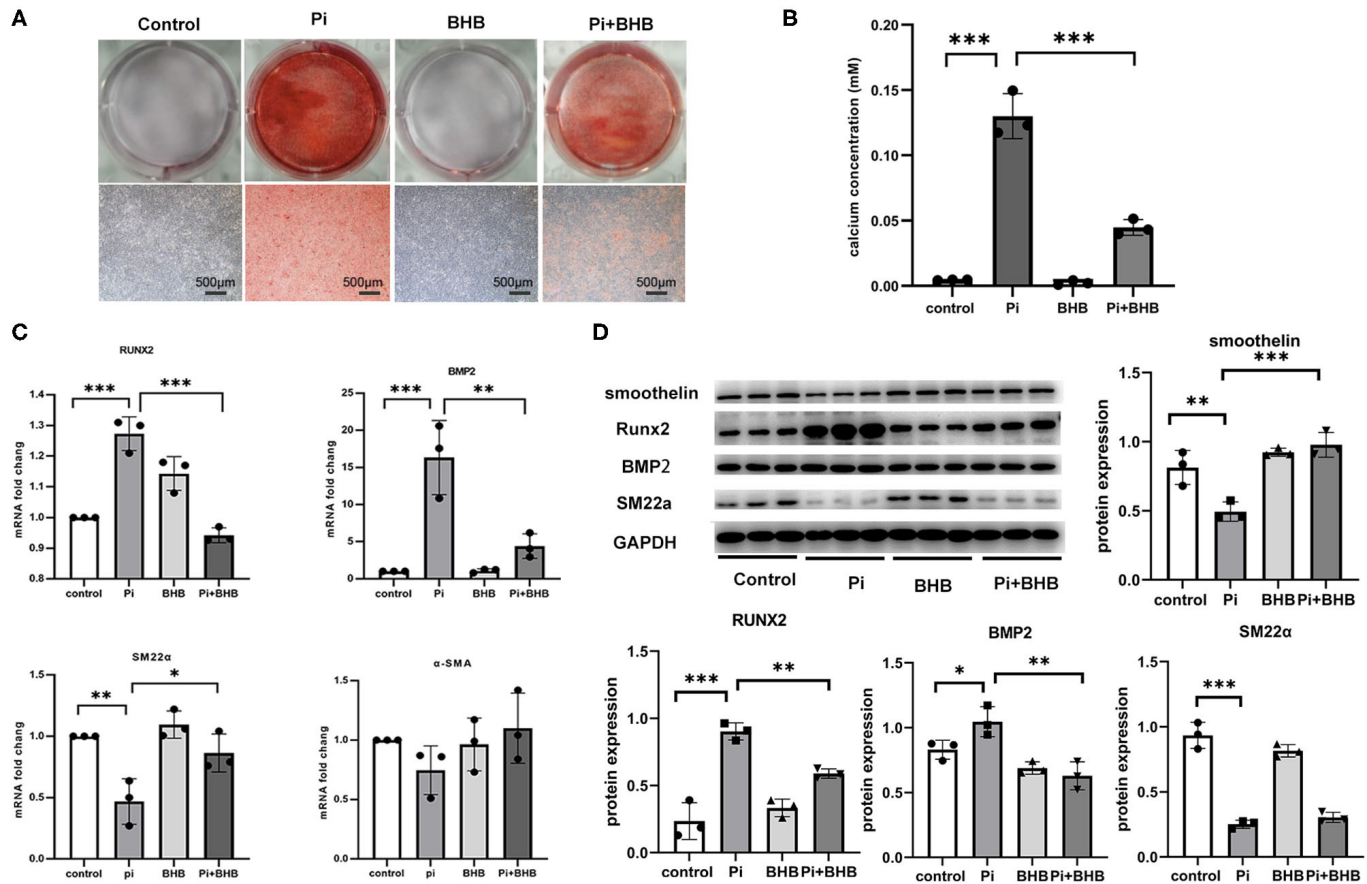
β-Hydroxybutyric acid (BHB) inhibited the osteogenic phenotypical transition of vascular smooth muscle cells (VSMCs). **(A)** Alizarin Red S staining of VSMCs. Primary VSMCs were treated with Pi (2.6 mM), BHB (4.0 mM), or Pi (2.6 mM) + BHB (4.0 mM) for 7 days. Scale bar: 500 μm. **(B)** Calcium density analysis examined the calcium concentration in supernatant collected from VSMCs. **(C)** qPCR detected the differences in the transcription levels of Runx2, BMP2, SM22α, and α-SMA. **(D)** Representative Western blot bands and semi-quantitative analysis of the protein expression of smoothelin, Runx2, BMP2, and SM22a. *P < 0.05; **P < 0.01; ***P < 0.001.

To further evaluate the effects of BHB on VC, we established calcification models with arterial rings and VSMCs and found that BHB co-incubation delayed the progression of the arterial calcification (Figure 2), and BHB intervention (2.6 mM Pi + 4.0 mM BHB) ameliorated VSMC calcification and decreased the calcium content in VSMCs (Figures 3A,B). Furthermore, with the analysis of RT-PCR and WB, the expressions of osteogenic markers, Runx2 and BMP2, were decreased in VSMCs treated with BHB intervention medium compared to cells treated with phosphate (2.6 mM Pi) alone, whereas BHB maintains the expressions of contractile proteins, smoothelin, and SM22α (Figures 3C,D). These results demonstrated that BHB may serve as an effective intervention for VC *via* regulating VSMC phenotypical transition.

### Autophagy Enhancement Induced by BHB Was Found to Inhibit VC

**Figure 4:**
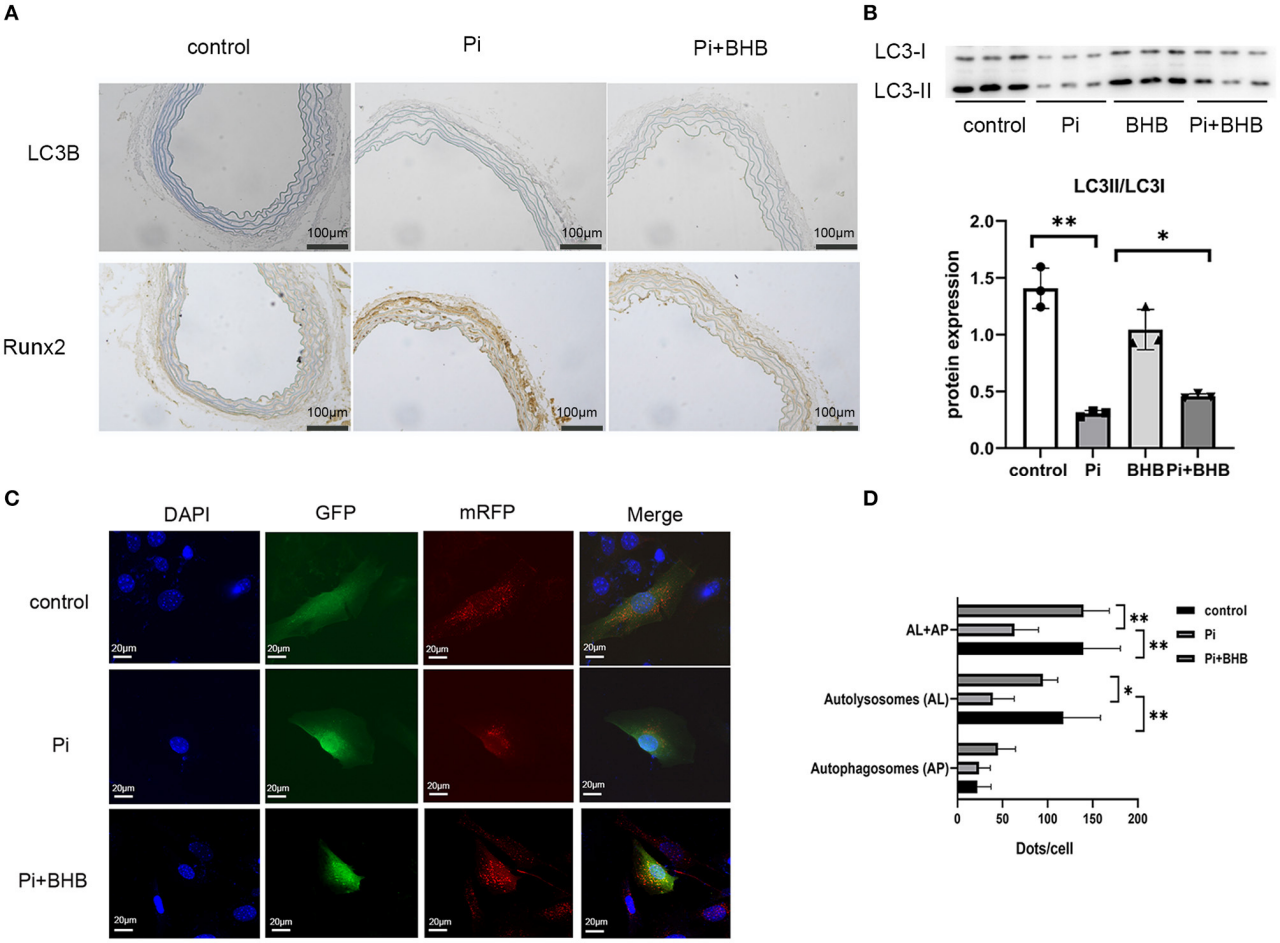
β-Hydroxybutyric acid-enhanced autophagy. **(A)** Immunohistochemical analysis detected the expression of LC3B and Runx2. Scale bar: 100 μm. **(B)** Representative Western blot bands and semi-quantitative analysis of LC3II/LC3-I. **(C,D)** Representative confocal images (scale bar, 20 μm) and quantitative analysis of mRFP-GFP-LC3 puncta in vascular smooth muscle cells. The bar graph shows the mean number of autophagosomes (yellow dots) and autolysosomes (red dots) per cell. *N* = 5 per group. *P < 0.05; **P < 0.01.

**Figure 5:**
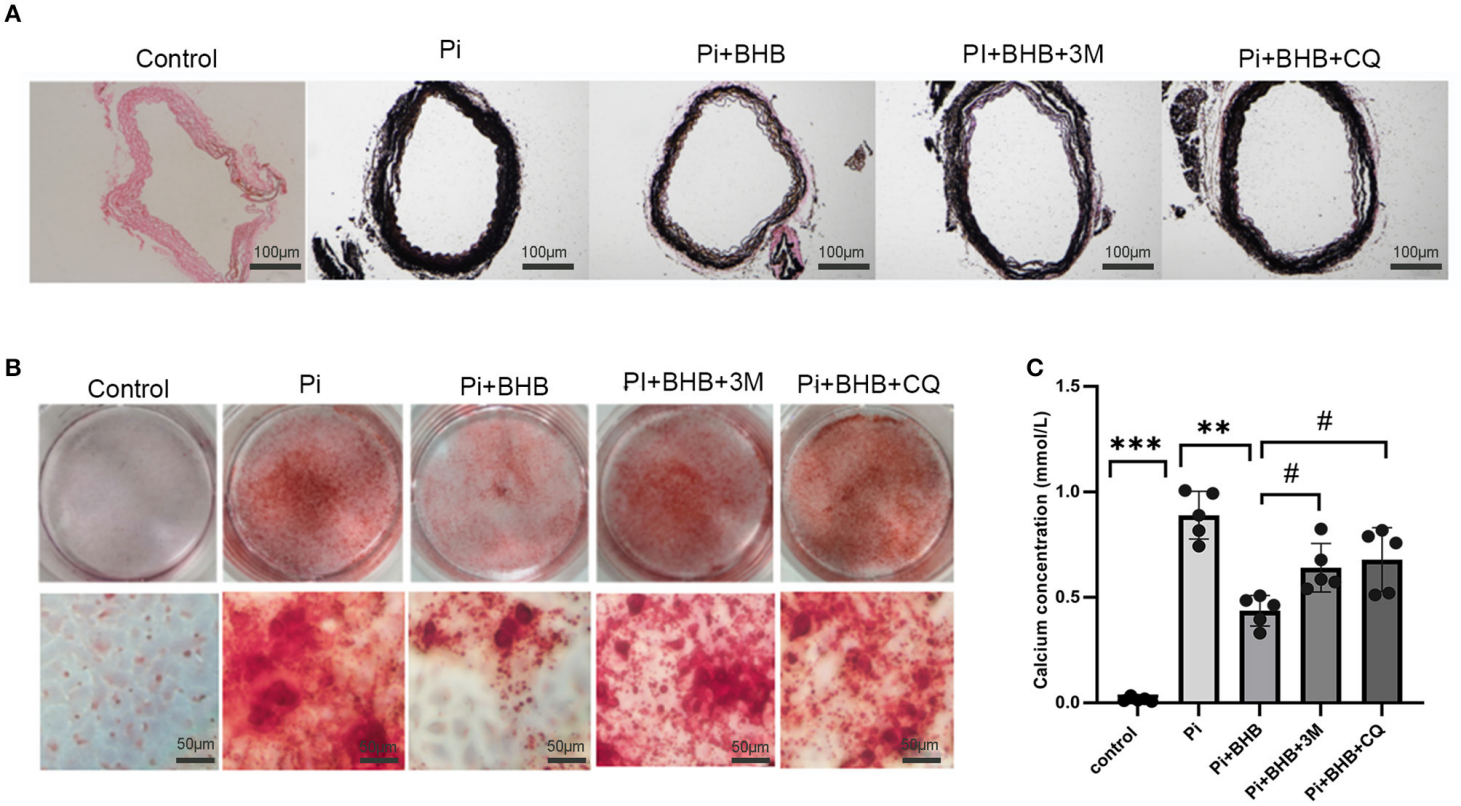
Autophagy inhibitors counteracted the protective effect of β-hydroxybutyric acid (BHB) on vascular calcification. **(A)** Von Kossa staining of arterial rings. **(B,C)** Alizarin Red S staining and calcium deposition analysis of vascular smooth muscle cells (VSMCs). Scale bar: 50 μm. *P < 0.05, **P < 0.01 vs. VSMCs treated with Pi alone; ^#^*P* < 0.05 vs. VSMCs treated with Pi+BHB.

To further investigate whether autophagy is the potential mechanism in BHB-mediated VC inhibition, this study performed several *ex vivo* and *in vitro* experiments. The results showed that BHB introduction postponed the progression of arterial calcification, enhanced the expression of autophagy-related protein LC3B, and upregulated the ratio of LC3II/LC3I (Figures 4A,B). To investigate the effects of BHB on autophagy flux, VSMCs were infected with mRFP-GFP-LC3 adenovirus (multiplicity of infection = 100). As a result, BHB administration induced a considerable increase in RFP-positive autolysosomes (red dots) in cells compared with those treated with high Pi alone (Figures 4C,D), while no differences were shown in GFP/RFP double-positive autophagosomes (yellow dots) between groups. Taken together, these results indicated that phosphate inhibits autophagy mainly by suppressing autophagic flux at late stage, while BHB offset this effect and promoted the fusion of autophagosomes with lysosomes to form autolysosomes. The inhibition of autophagy in VSMCs counteracted the protective effect of BHB on arterial calcification and increased the calcium content in VSMCs (Figure 5). In conclusion, the findings demonstrated that the BHB-induced autophagy enhancement may be a potential mechanism that inhibited the development of VC.

## Discussion

The aim of this study was to determine whether BHB ameliorated arterial calcification and to investigate the potential mechanism related to autophagy. The major findings of our present study are as follows: (1) BHB was negatively correlated with VC and mediated the effects of MAs on VC, (2) BHB postponed arterial calcification and inhibited the osteogenic phenotype transdifferentiation of VSMCs induced by high phosphate, and (3) autophagy enhancement promoted by BHB in VSMCs was found to contribute to the inhibition of arterial calcification. For the first time, this study demonstrated the role and effect of BHB in VC and MAs. Furthermore, the results may provide a potential interventional target for the prevention of VC.

MAs were common in the aging population. Data from the National Health and Nutrition Examination Survey showed that the prevalence of MAs was about 46.7% among subjects of age >60 years (30). It has been well-accepted that aging is a powerful risk factor for VC and METs (31). In accordance, interventions toward aging or MAs may possess potency in preventing VC (32, 33). We confirmed, with a univariate linear regression analysis, that serum BHB levels were negatively related with TAC severities. Moreover, after including various potential variables into the regression model, we showed that BHB was still an independent protective factor for severe TAC. To figure out whether BHB transmitted the effects of MAs to VC, we performed a mediation analysis, and the result confirmed that BHB was the mediator (intervening variable), indicating that BHB is a potential target to regulate metabolic conditions and therefore affects the pathogenesis of VC. However, the underlying effects and mechanisms of BHB on VC remain unclear.

Accumulating data from observational and randomized clinical trials indicated that CR might improve cardiovascular health and metabolic status (34, 35). CR upregulates the production of ketone bodies, in which BHB accounted for more than 70%, a multifunctional metabolic substance which could be physiologically elevated under glucose deprivation. Recent studies suggested that BHB played an important role in delaying aging and improving metabolic conditions (18, 24, 29, 36).

Exercise and calorie restriction diets are believed to improve metabolic status. The metabolic status amelioration may be linked to the upregulation of BHB. Interestingly, sodium–glucose cotransporter 2 inhibitors, such as dapagliflozin, an effective drug for T2DM, could induce mild ketonemia and downregulate serum glucose, reduce body weight, and blood pressure (37). BHB is the most promising effector during the process, for researchers have confirmed that the administration of BHB precursor could attenuate hypertension and protect against heart diseases (29).

Moreover, BHB takes a part in maintaining the functions of the cardiovascular system—for example, BHB could delay vascular aging, improve cardiac ischemia/reperfusion injury, and inhibit inflammation (38, 39). However, it remains unclear whether BHB affects VC, and the underlying mechanism needs to be elucidated. In this study, we established vascular calcification models and evaluate the effects of BHB on VC. The Von Kossa staining of the aorta rings showed that BHB significantly alleviated arterial calcification, while the Alizarin Red S staining demonstrated that BHB reduced the calcium content of VSMCs. Moreover, we further demonstrated that BHB inhibited the osteogenic phenotype transdifferentiation of VSMCs, which was characterized by the upregulation of osteogenic phenotype markers (RUNX2 and BMP2) and downregulation of contractile phenotype markers (smoothelin and SM22a).

However, the underlying mechanisms in the regulation of BHB on VSMC calcification need further studies. It has been proven that autophagy, an evolutionarily conserved mechanism linking to several cellular pathways, impacts VSMC survival and functions. Dai et al. demonstrated that the inhibition of autophagy enhanced the phosphate-induced matrix vesicle release, thereby exacerbating VSMC calcification (40). Otherwise, BHB was found to enhance autophagy regulated by the histone acetylation of specific genes and improve proteostasis through increased chaperone expression (41). Thus, it is meaningful to further explore whether BHB can promote autophagy in VSMCs to protect against osteogenic phenotype transdifferentiation, resulting in inhibiting VC. In the present study, BHB enhanced the expression of autophagy-related protein LC3B and promoted the formation of autolysosomes. The results may raise the perspective attention for the intervention of BHB on inhibiting VC.

Our study has some limitations which must be appreciated. Firstly, the clinical study was retrospective; confounding factors or selection bias may have affected our findings. A prospective study for the effect of BHB on VC should be considered in the future. Secondly, the current project should be further validated in BHB-knock-out animal models; however, the model is not available because BHB is important for animal survival.

In conclusion, we identified for the first time that BHB mediated the correlation between MAs and VC; then, we further demonstrated BHB could promote autophagy to inhibit VC. Our present study provides a novel insight into the potential of BHB as an effective strategy for the treatment of arterial calcification.

## Perspectives

This is the first study to demonstrate that BHB mediated the correlation between MAs and arterial calcification. In addition, the enhancement of autophagy in VSMCs induced by BHB treatment may be an effective intervention target to ameliorate arterial calcification and delay aging.

## Data Availability Statement

The original contributions presented in the study are included in the article/supplementary material, further inquiries can be directed to the corresponding author/s.

## Ethics Statement

The studies involving human participants were reviewed and approved by Ethics Committee of the Eighth Affiliated Hospital, Sun Yat-sen University. The patients/participants provided their written informed consent to participate in this study. The animal study was reviewed and approved by Ethics Committee of the Eighth Affiliated Hospital, Sun Yat-sen University.

## Author Contributions

JL: funding acquisition, writing—original draft, and writing—review and editing. JH: conceptualization, data curation, formal analysis, and investigation. WH: data curation, formal analysis, and investigation. GS: computerized tomographic analysis. JC: supervision. HH: funding acquisition and supervision. All authors contributed to the article and approved the submitted version.

## Conflict of Interest

The authors declare that the research was conducted in the absence of any commercial or financial relationships that could be construed as a potential conflict of interest.

## Publisher's Note

All claims expressed in this article are solely those of the authors and do not necessarily represent those of their affiliated organizations, or those of the publisher, the editors and the reviewers. Any product that may be evaluated in this article, or claim that may be made by its manufacturer, is not guaranteed or endorsed by the publisher.
